# Tris(2-{[2-(4-meth­oxy­phen­yl)eth­yl]imino­meth­yl}phenolato-κ^2^ *N*,*O* ^1^)cobalt(III)

**DOI:** 10.1107/S1600536812023033

**Published:** 2012-05-26

**Authors:** Ali Ourari, Yasmina Ouennoughi, Sofiane Bouacida

**Affiliations:** a Laboratoire d’Electrochimie, d’Ingénierie moléculaire et de Catalyse Redox (LEIMCR), Faculté des Sciences de l’Ingénieur, Université Farhat Abbas, Sétif 19000, Algeria; b Unité de Recherche de Chimie de l’Environnement et Moléculaire Structurale, CHEMS, Université Mentouri–Constantine, 25000 Algeria

## Abstract

In the title compound, [Co(C_16_H_16_NO_2_)_3_], the Co^III^ atom is six-coordinated in an irregular octa­hedral geometry by three *N*,*O*-chelating 2-{[2-(4-meth­oxy­phen­yl)eth­yl]imino­meth­yl}phenolate groups. One of the three meth­oxy group is disordered over two sets of sites with an occupancy ratio of 0.768 (5):0.232 (5). The crystal packing can be described by alternating zigzag layers of organic ligands and CoN_3_O_3_ octa­hedra along the *c* axis. There are no classical hydrogen bonds in the structure, but C—H⋯π inter­actions occur.

## Related literature

For the synthesis and applications of similar compounds and derivates see: Ourari *et al.* (2008 ▶, 2011 ▶); Van Praag (1981 ▶); Yu *et al.* (2003 ▶). 
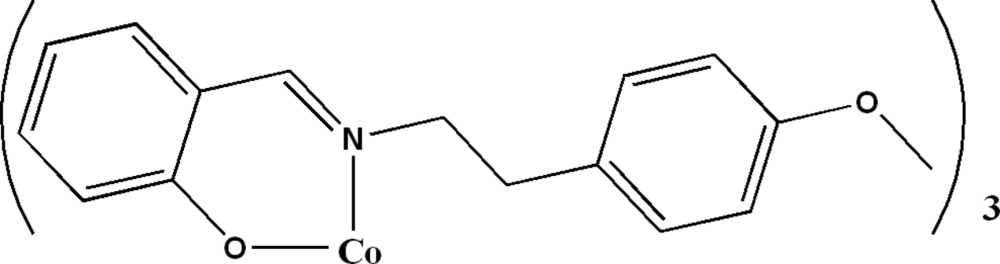


## Experimental

### 

#### Crystal data

[Co(C_16_H_16_NO_2_)_3_]
*M* *_r_* = 821.82
Orthorhombic, 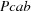

*a* = 15.9798 (5) Å
*b* = 19.2968 (6) Å
*c* = 27.4387 (9) Å
*V* = 8461.0 (5) Å^3^
*Z* = 8
Mo *K*α radiation
μ = 0.46 mm^−1^
*T* = 150 K
0.51 × 0.15 × 0.09 mm

#### Data collection

Bruker APEXII diffractometer
Absorption correction: multi-scan (*SADABS*; Sheldrick, 2002 ▶) *T*_min_ = 0.789, *T*_max_ = 0.960
39521 measured reflections
9652 independent reflections
6226 reflections with *I* > 2σ(*I*)
*R*_int_ = 0.052

#### Refinement

*R*[*F* ^2^ > 2σ(*F* ^2^)] = 0.056
*wR*(*F* ^2^) = 0.125
*S* = 1.01
9652 reflections
531 parameters
H-atom parameters constrained
Δρ_max_ = 1.10 e Å^−3^
Δρ_min_ = −0.30 e Å^−3^

### 

Data collection: *APEX2* (Bruker, 2001 ▶); cell refinement: *SAINT* (Bruker, 2001 ▶); data reduction: *SAINT*; program(s) used to solve structure: *SIR2002* (Burla *et al.*, 2005 ▶); program(s) used to refine structure: *SHELXL97* (Sheldrick, 2008 ▶); molecular graphics: *ORTEP-3 for Windows* (Farrugia, 1997 ▶) and *DIAMOND* (Brandenburg & Berndt, 2001 ▶); software used to prepare material for publication: *WinGX* (Farrugia, 1999 ▶) and *CRYSCAL* (T. Roisnel, local program).

## Supplementary Material

Crystal structure: contains datablock(s) global, I. DOI: 10.1107/S1600536812023033/bq2360sup1.cif

Structure factors: contains datablock(s) I. DOI: 10.1107/S1600536812023033/bq2360Isup2.hkl

Additional supplementary materials:  crystallographic information; 3D view; checkCIF report

## Figures and Tables

**Table 1:** Selected bond lengths (Å)

Co1—O1	1.9054 (16)
Co1—N9	1.952 (2)
Co1—O21	1.8791 (17)
Co1—N29	1.9512 (19)
Co1—O41	1.8955 (18)
Co1—N49	1.944 (2)

**Table 2:** Hydrogen-bond geometry (Å, °) *Cg*1 and *Cg*2 are the centroids of the C52–C57 and C42–C47 rings, respectively.

*D*—H⋯*A*	*D*—H	H⋯*A*	*D*⋯*A*	*D*—H⋯*A*
C4—H4⋯*Cg*1^i^	0.95	2.56	3.475 (3)	162
C30—H30*B*⋯*Cg*2^ii^	0.99	2.91	3.838 (3)	157

Symmetry codes: (i) 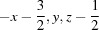; (ii) 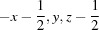.

**Fig. 1.**
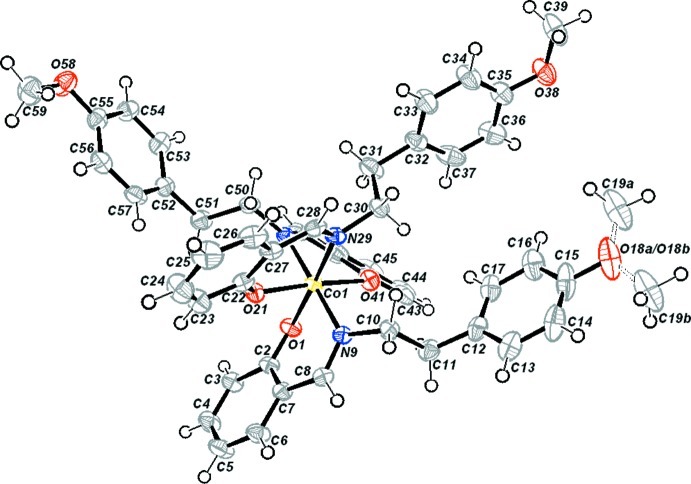
The molecular geometry of (I) with the atom-labeling scheme. Displacement ellipsoids are drawn at the 50% probability level.

**Fig. 2.**
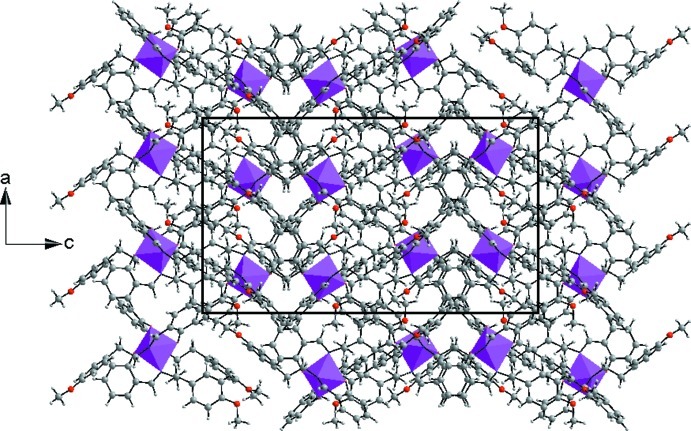
Packing diagram of (I) viewed down b-axis showing altering layers in zigzag.

## supplementary crystallographic information

### Comment

The *O*-methyltyramine (4-Mthoxyphenyl)ethlamine is an important pharmaceutical product which is metabolized by monoamineoxidases. This compound may be considered a precursor for neurotransmitter in the central nervous system (Van Praag, 1981) and neurohormone in the blood circulation. This amine reacted, in ethanolic solution, with salicylaldehyde to give *N*-Salicylidene(4-methoxyphenylethylamine) as bidentate Schiff base ligand (HL). This ligand, dissolved in absolute ethanol with a cobalt salt leads to the formation of its corresponding cobalt complex (Co(III)-3*L*). The electrocatalytic performaces of this cobalt complex towards the oxidation saturated hydrocarbons or epoxidation of olefins *via* cytochrome P450 model (Yu *et al.*, 2003) by using molecular oxygen is now under progress in our laboratory (Ourari *et al.*, 2008; Ourari *et al.*, 2011). Thus, we report here the synthesis of title compound and its crystal structure. The molecular geometry of (I), and the atomic numbering used, is illustrated in Fig. 1. The Co atom is six coordinated in a irregular octahedral geometry by three (Salicylidene-(4-Methoxyphenyl)ethylamine)groups, while each one is in bidentate chelating coordination with N and O atoms. The bond lengths for co-ordination cobalt sphere is ranging from 1.8791 (17) to 1.9054 (16) Å for Co—O distances and from 1.944 (2) to 1.952 (2) Å for Co—N distances (Table 1). The crystal packing in the title structure can be described by alterning layers in zigzag of organic ligand and CoN_3_O_3_ octahedral along the *c* axis (Fig. 2). C—H···π interactions consolidate the stabilization (Table 2).

### Experimental

151 mg (1 mmol) of (4-methoxyphenyl)ethylamine were dissolved in 8 ml of absolute ethanol and placed in three necked flask surmounted by a condenser. Then, an ethanolic solution of 122 mg (1 mmol) of salicylaldehyde (5 ml) was added drop wise to the previous solution. After addition of the first drops under stirring, the solution turns to the yellow color and then is heated to reflux for 2 h. 238 mg of cobalt chloride hexahydrated (CoCl_2_,6H_2_O) were also dissolved in 8 ml of absolute ethanol and added to the ligand solution. This mixture was refluxed again under stirring for 2 other hours to give finally a solid which is recovered by filtration, washed several times with small portions of distilled water. The solid (crystals) was dried in vaccuo in presence of CaCl_2_ to yield 168 mg (61%).

### Refinement

H atoms were localized on Fourier maps but introduced in calculated positions and treated as riding on their parent atoms (C) with C—H = 0.98 Å (methyl), 0.99 Å (methylene) and 0.95 Å (aromatic) with *U*_iso_(H) = 1.2*U*_eq_(C_aromatic_, C_methylene_ and C_methyl_) and *U*_iso_(H) = 1.5*U*_eq_(C_methyl_).

One of three methoxy group is disordered in two sites with carbon atoms C19A and C19B (77/23).

### Crystal data

**Table:**

[Co(C_16_H_16_NO_2_)_3_]	*F*(000) = 3456
*M**_r_* = 821.82	*D*_x_ = 1.29 Mg m^−^^3^
Orthorhombic, *P**c**a**b*	Mo *K*α radiation, λ = 0.71073 Å
Hall symbol: -P 2bc 2ac	Cell parameters from 8141 reflections
*a* = 15.9798 (5) Å	θ = 2.5–27.1°
*b* = 19.2968 (6) Å	µ = 0.46 mm^−^^1^
*c* = 27.4387 (9) Å	*T* = 150 K
*V* = 8461.0 (5) Å^3^	Prism, black
*Z* = 8	0.51 × 0.15 × 0.09 mm

### Data collection

**Table:**

Bruker APEXII diffractometer	6226 reflections with *I* > 2σ(*I*)
Graphite monochromator	*R*_int_ = 0.052
CCD rotation images, thin slices scans	θ_max_ = 27.5°, θ_min_ = 3.0°
Absorption correction: multi-scan (*SADABS*; Sheldrick, 2002)	*h* = −20→14
*T*_min_ = 0.789, *T*_max_ = 0.960	*k* = −17→24
39521 measured reflections	*l* = −32→35
9652 independent reflections	

### Refinement

**Table:**

Refinement on *F*^2^	Primary atom site location: structure-invariant direct methods
Least-squares matrix: full	Secondary atom site location: difference Fourier map
*R*[*F*^2^ > 2σ(*F*^2^)] = 0.056	Hydrogen site location: inferred from neighbouring sites
*wR*(*F*^2^) = 0.125	H-atom parameters constrained
*S* = 1.01	*w* = 1/[σ^2^(*F*_o_^2^) + (0.0487*P*)^2^ + 5.3662*P*] where *P* = (*F*_o_^2^ + 2*F*_c_^2^)/3
9652 reflections	(Δ/σ)_max_ = 0.002
531 parameters	Δρ_max_ = 1.10 e Å^−^^3^
0 restraints	Δρ_min_ = −0.30 e Å^−^^3^

### Special details

**Table:**

Geometry. All e.s.d.'s (except the e.s.d. in the dihedral angle between two l.s. planes) are estimated using the full covariance matrix. The cell e.s.d.'s are taken into account individually in the estimation of e.s.d.'s in distances, angles and torsion angles; correlations between e.s.d.'s in cell parameters are only used when they are defined by crystal symmetry. An approximate (isotropic) treatment of cell e.s.d.'s is used for estimating e.s.d.'s involving l.s. planes.
Refinement. Refinement of *F*^2^ against ALL reflections. The weighted *R*-factor *wR* and goodness of fit *S* are based on *F*^2^, conventional *R*-factors *R* are based on *F*, with *F* set to zero for negative *F*^2^. The threshold expression of *F*^2^ > σ(*F*^2^) is used only for calculating *R*-factors(gt) *etc*. and is not relevant to the choice of reflections for refinement. *R*-factors based on *F*^2^ are statistically about twice as large as those based on *F*, and *R*- factors based on ALL data will be even larger.

### Fractional atomic coordinates and isotropic or equivalent isotropic displacement parameters (Å^2^)

**Table:**

	*x*	*y*	*z*	*U*_iso_*/*U*_eq_	Occ. (<1)
O1	0.56611 (9)	0.21320 (8)	0.15417 (7)	0.0262 (4)	
C2	0.54271 (14)	0.15914 (13)	0.17995 (10)	0.0242 (6)	
C3	0.47832 (14)	0.16585 (14)	0.21439 (10)	0.0289 (6)	
H3	0.4531	0.2099	0.2192	0.035*	
C4	0.45099 (16)	0.11071 (14)	0.24119 (11)	0.0359 (7)	
H4	0.4075	0.1172	0.2643	0.043*	
C5	0.48579 (18)	0.04492 (15)	0.23521 (12)	0.0418 (8)	
H5	0.4668	0.0069	0.2543	0.05*	
C6	0.54782 (17)	0.03625 (14)	0.20135 (11)	0.0356 (7)	
H6	0.5711	−0.0085	0.1965	0.043*	
C7	0.57773 (14)	0.09216 (13)	0.17377 (10)	0.0265 (6)	
C8	0.64112 (15)	0.08016 (13)	0.13742 (10)	0.0271 (6)	
H8	0.6505	0.0335	0.1278	0.032*	
N9	0.68604 (11)	0.12686 (10)	0.11689 (8)	0.0228 (5)	
C10	0.73736 (14)	0.10433 (14)	0.07502 (10)	0.0272 (6)	
H10A	0.7635	0.0591	0.0825	0.033*	
H10B	0.7826	0.1384	0.0693	0.033*	
C11	0.68374 (16)	0.09763 (15)	0.02874 (10)	0.0347 (7)	
H11A	0.6558	0.0519	0.0287	0.042*	
H11B	0.6397	0.1337	0.0292	0.042*	
C12	0.73438 (16)	0.10498 (16)	−0.01714 (10)	0.0357 (7)	
C13	0.7742 (2)	0.04917 (18)	−0.03915 (12)	0.0498 (8)	
H13	0.7666	0.0039	−0.0263	0.06*	
C14	0.8251 (2)	0.0585 (2)	−0.07970 (12)	0.0592 (10)	
H14	0.8523	0.0197	−0.094	0.071*	
C15	0.8363 (2)	0.1236 (2)	−0.09922 (11)	0.0552 (10)	
C16	0.79647 (19)	0.18006 (19)	−0.07850 (12)	0.0497 (8)	
H16	0.8036	0.2252	−0.0917	0.06*	
C17	0.74595 (17)	0.16956 (17)	−0.03799 (11)	0.0406 (7)	
H17	0.7182	0.2083	−0.024	0.049*	
O18A	0.88982 (17)	0.13000 (17)	−0.13833 (9)	0.0768 (8)	0.768 (5)
C19A	0.9198 (3)	0.1989 (3)	−0.15299 (19)	0.0785 (17)	0.768 (5)
H19A	0.8733	0.2255	−0.1668	0.118*	0.768 (5)
H19B	0.964	0.1938	−0.1775	0.118*	0.768 (5)
H19C	0.9421	0.2232	−0.1244	0.118*	0.768 (5)
O18B	0.88982 (17)	0.13000 (17)	−0.13833 (9)	0.0768 (8)	0.232 (5)
C19B	0.9006 (10)	0.0661 (10)	−0.1666 (6)	0.0785 (17)	0.232 (5)
H19D	0.9337	0.0329	−0.1477	0.118*	0.232 (5)
H19E	0.9297	0.0767	−0.1972	0.118*	0.232 (5)
H19F	0.8457	0.0461	−0.1739	0.118*	0.232 (5)
O21	0.70085 (9)	0.19705 (9)	0.20255 (6)	0.0246 (4)	
C22	0.77056 (15)	0.17339 (13)	0.22095 (10)	0.0252 (6)	
C23	0.76778 (17)	0.14308 (14)	0.26742 (11)	0.0358 (7)	
H23	0.7153	0.1372	0.2832	0.043*	
C24	0.83921 (19)	0.12180 (17)	0.29045 (12)	0.0484 (8)	
H24	0.8358	0.1019	0.322	0.058*	
C25	0.91760 (18)	0.12910 (16)	0.26782 (13)	0.0447 (8)	
H25	0.9672	0.1151	0.2842	0.054*	
C26	0.92159 (16)	0.15656 (14)	0.22208 (11)	0.0345 (7)	
H26	0.9744	0.1606	0.2065	0.041*	
C27	0.84910 (15)	0.17899 (12)	0.19750 (10)	0.0249 (6)	
C28	0.85865 (15)	0.21238 (12)	0.15104 (10)	0.0254 (6)	
H28	0.9143	0.218	0.1395	0.031*	
N29	0.79997 (12)	0.23556 (10)	0.12334 (8)	0.0235 (5)	
C30	0.82819 (16)	0.26863 (14)	0.07743 (10)	0.0309 (6)	
H30A	0.7938	0.2505	0.0503	0.037*	
H30B	0.8869	0.2549	0.0712	0.037*	
C31	0.82291 (18)	0.34717 (15)	0.07722 (12)	0.0417 (7)	
H31A	0.7638	0.3621	0.0799	0.05*	
H31B	0.8542	0.3664	0.1053	0.05*	
C32	0.86010 (17)	0.37328 (14)	0.03002 (11)	0.0338 (7)	
C33	0.94358 (18)	0.38949 (17)	0.02659 (11)	0.0450 (8)	
H33	0.9775	0.3864	0.0549	0.054*	
C34	0.97996 (17)	0.41015 (18)	−0.01690 (11)	0.0464 (8)	
H34	1.0377	0.4217	−0.0179	0.056*	
C35	0.93267 (17)	0.41393 (14)	−0.05816 (11)	0.0352 (7)	
C36	0.84899 (19)	0.39669 (17)	−0.05613 (12)	0.0480 (8)	
H36	0.8157	0.3984	−0.0848	0.058*	
C37	0.81389 (18)	0.37702 (16)	−0.01253 (13)	0.0463 (8)	
H37	0.7561	0.3656	−0.0116	0.056*	
O38	0.96291 (13)	0.43430 (12)	−0.10290 (8)	0.0483 (6)	
C39	1.04959 (19)	0.4520 (2)	−0.10525 (13)	0.0566 (10)	
H39A	1.0619	0.4879	−0.0811	0.085*	
H39B	1.0629	0.4693	−0.1379	0.085*	
H39C	1.0835	0.4108	−0.0984	0.085*	
O41	0.65062 (10)	0.24732 (9)	0.07303 (6)	0.0283 (4)	
C42	0.58993 (15)	0.29141 (14)	0.06451 (10)	0.0281 (6)	
C43	0.54282 (16)	0.28446 (16)	0.02098 (11)	0.0373 (7)	
H43	0.5548	0.2478	−0.0011	0.045*	
C44	0.47934 (17)	0.33108 (18)	0.01056 (12)	0.0472 (9)	
H44	0.4473	0.325	−0.0183	0.057*	
C45	0.46127 (17)	0.38620 (18)	0.04092 (13)	0.0469 (9)	
H45	0.4176	0.4177	0.033	0.056*	
C46	0.50732 (16)	0.39484 (16)	0.08281 (12)	0.0410 (8)	
H46	0.4959	0.433	0.1036	0.049*	
C47	0.57117 (15)	0.34779 (14)	0.09524 (11)	0.0304 (6)	
C48	0.62041 (15)	0.36157 (13)	0.13794 (10)	0.0294 (6)	
H48	0.614	0.4058	0.1526	0.035*	
N49	0.67229 (12)	0.31964 (11)	0.15815 (8)	0.0257 (5)	
C50	0.72310 (15)	0.34684 (14)	0.19892 (10)	0.0282 (6)	
H50A	0.7431	0.3939	0.1904	0.034*	
H50B	0.7728	0.3169	0.2033	0.034*	
C51	0.67522 (16)	0.35037 (15)	0.24693 (10)	0.0351 (7)	
H51A	0.6255	0.3805	0.243	0.042*	
H51B	0.6557	0.3034	0.256	0.042*	
C52	0.73046 (15)	0.37882 (15)	0.28679 (10)	0.0312 (6)	
C53	0.74935 (16)	0.44964 (15)	0.28894 (11)	0.0337 (7)	
H53	0.7248	0.4801	0.2658	0.04*	
C54	0.80241 (16)	0.47641 (14)	0.32355 (11)	0.0339 (7)	
H54	0.8143	0.5246	0.324	0.041*	
C55	0.83868 (16)	0.43290 (14)	0.35790 (10)	0.0319 (6)	
C56	0.82222 (17)	0.36240 (15)	0.35614 (10)	0.0347 (7)	
H56	0.8473	0.332	0.3791	0.042*	
C57	0.76894 (17)	0.33646 (15)	0.32061 (11)	0.0352 (7)	
H57	0.7586	0.288	0.3195	0.042*	
O58	0.88894 (12)	0.46474 (10)	0.39157 (8)	0.0427 (5)	
C59	0.9264 (2)	0.42236 (17)	0.42778 (12)	0.0490 (8)	
H59A	0.8826	0.398	0.446	0.074*	
H59B	0.9589	0.4513	0.4502	0.074*	
H59C	0.9634	0.3885	0.4122	0.074*	
Co1	0.681248 (19)	0.223520 (17)	0.137637 (12)	0.02173 (10)	

### Atomic displacement parameters (Å^2^)

**Table:**

	*U*^11^	*U*^22^	*U*^33^	*U*^12^	*U*^13^	*U*^23^
O1	0.0146 (8)	0.0290 (10)	0.0349 (10)	0.0026 (7)	0.0011 (7)	0.0048 (9)
C2	0.0135 (11)	0.0284 (14)	0.0307 (15)	−0.0016 (10)	−0.0044 (11)	0.0034 (12)
C3	0.0184 (12)	0.0315 (15)	0.0367 (16)	0.0047 (11)	0.0034 (11)	0.0022 (13)
C4	0.0265 (14)	0.0399 (17)	0.0414 (18)	0.0005 (12)	0.0133 (13)	0.0008 (14)
C5	0.0402 (16)	0.0314 (16)	0.054 (2)	−0.0045 (13)	0.0187 (15)	0.0049 (15)
C6	0.0326 (15)	0.0265 (15)	0.0477 (19)	0.0001 (12)	0.0101 (14)	−0.0005 (14)
C7	0.0179 (12)	0.0306 (15)	0.0310 (15)	−0.0011 (11)	0.0022 (11)	−0.0013 (12)
C8	0.0226 (13)	0.0254 (14)	0.0332 (15)	0.0014 (11)	−0.0020 (12)	−0.0021 (13)
N9	0.0172 (10)	0.0244 (11)	0.0268 (11)	0.0015 (9)	0.0008 (9)	0.0002 (10)
C10	0.0189 (12)	0.0318 (15)	0.0310 (15)	−0.0003 (10)	0.0028 (11)	−0.0022 (12)
C11	0.0264 (14)	0.0424 (17)	0.0353 (16)	−0.0031 (12)	−0.0030 (13)	−0.0067 (14)
C12	0.0276 (14)	0.0503 (19)	0.0292 (16)	0.0046 (13)	−0.0098 (12)	−0.0080 (15)
C13	0.054 (2)	0.053 (2)	0.042 (2)	0.0077 (16)	−0.0042 (16)	−0.0118 (17)
C14	0.067 (2)	0.073 (3)	0.037 (2)	0.027 (2)	0.0003 (18)	−0.0147 (19)
C15	0.0472 (19)	0.093 (3)	0.0257 (17)	0.0263 (19)	−0.0027 (15)	−0.0039 (19)
C16	0.0430 (18)	0.071 (2)	0.0354 (18)	0.0137 (16)	−0.0064 (15)	0.0054 (17)
C17	0.0326 (15)	0.057 (2)	0.0324 (17)	0.0089 (14)	−0.0030 (13)	−0.0045 (16)
O18A	0.086 (2)	0.108 (2)	0.0364 (14)	0.0288 (17)	0.0159 (13)	0.0074 (16)
C19A	0.054 (3)	0.126 (5)	0.055 (3)	0.021 (3)	0.015 (2)	0.033 (3)
O18B	0.086 (2)	0.108 (2)	0.0364 (14)	0.0288 (17)	0.0159 (13)	0.0074 (16)
C19B	0.054 (3)	0.126 (5)	0.055 (3)	0.021 (3)	0.015 (2)	0.033 (3)
O21	0.0179 (8)	0.0302 (10)	0.0258 (10)	0.0022 (7)	0.0000 (7)	0.0011 (8)
C22	0.0229 (13)	0.0206 (13)	0.0319 (15)	0.0028 (10)	−0.0034 (11)	−0.0016 (12)
C23	0.0294 (14)	0.0393 (17)	0.0387 (17)	0.0042 (12)	−0.0011 (13)	0.0094 (14)
C24	0.0456 (18)	0.055 (2)	0.0445 (19)	0.0074 (15)	−0.0084 (16)	0.0196 (17)
C25	0.0317 (16)	0.0432 (18)	0.059 (2)	0.0105 (13)	−0.0148 (15)	0.0097 (17)
C26	0.0206 (13)	0.0287 (15)	0.054 (2)	0.0063 (11)	−0.0051 (13)	0.0003 (14)
C27	0.0217 (12)	0.0166 (13)	0.0365 (16)	0.0031 (10)	−0.0026 (11)	−0.0013 (12)
C28	0.0175 (12)	0.0215 (14)	0.0372 (16)	−0.0007 (10)	0.0034 (11)	−0.0089 (12)
N29	0.0177 (10)	0.0224 (12)	0.0305 (12)	−0.0001 (8)	0.0019 (9)	−0.0012 (9)
C30	0.0238 (13)	0.0364 (16)	0.0325 (15)	−0.0027 (11)	0.0083 (12)	−0.0011 (13)
C31	0.0409 (16)	0.0329 (16)	0.051 (2)	−0.0012 (13)	0.0196 (15)	0.0023 (15)
C32	0.0320 (15)	0.0266 (15)	0.0427 (18)	0.0022 (12)	0.0084 (14)	0.0039 (13)
C33	0.0323 (16)	0.068 (2)	0.0350 (17)	−0.0059 (15)	−0.0024 (14)	0.0103 (17)
C34	0.0226 (14)	0.074 (2)	0.0425 (19)	−0.0036 (14)	−0.0015 (14)	0.0145 (17)
C35	0.0337 (15)	0.0351 (16)	0.0367 (17)	0.0058 (12)	0.0005 (13)	0.0094 (14)
C36	0.0376 (16)	0.055 (2)	0.052 (2)	−0.0044 (15)	−0.0138 (16)	0.0197 (17)
C37	0.0260 (14)	0.0466 (19)	0.066 (2)	−0.0029 (13)	−0.0045 (16)	0.0163 (17)
O38	0.0458 (12)	0.0626 (15)	0.0363 (12)	0.0010 (10)	0.0010 (10)	0.0187 (11)
C39	0.0455 (19)	0.078 (3)	0.047 (2)	0.0140 (17)	0.0183 (16)	0.0168 (19)
O41	0.0248 (9)	0.0327 (10)	0.0275 (10)	0.0033 (8)	−0.0005 (8)	0.0042 (9)
C42	0.0191 (12)	0.0336 (15)	0.0316 (15)	−0.0032 (11)	0.0043 (11)	0.0121 (13)
C43	0.0273 (14)	0.0494 (19)	0.0352 (16)	−0.0080 (13)	−0.0016 (12)	0.0179 (15)
C44	0.0251 (15)	0.067 (2)	0.049 (2)	−0.0090 (15)	−0.0084 (14)	0.0311 (19)
C45	0.0226 (14)	0.053 (2)	0.066 (2)	0.0043 (14)	0.0025 (15)	0.0322 (19)
C46	0.0255 (14)	0.0424 (18)	0.055 (2)	0.0022 (12)	0.0064 (14)	0.0216 (16)
C47	0.0178 (12)	0.0335 (15)	0.0399 (17)	0.0001 (11)	0.0041 (12)	0.0147 (14)
C48	0.0238 (13)	0.0254 (14)	0.0390 (16)	0.0025 (11)	0.0118 (12)	0.0029 (13)
N49	0.0184 (10)	0.0272 (12)	0.0316 (12)	−0.0012 (9)	0.0053 (9)	0.0007 (10)
C50	0.0212 (13)	0.0260 (14)	0.0373 (16)	−0.0021 (11)	0.0043 (12)	−0.0045 (12)
C51	0.0254 (13)	0.0379 (16)	0.0420 (17)	−0.0025 (12)	0.0097 (13)	−0.0077 (14)
C52	0.0232 (13)	0.0377 (16)	0.0328 (16)	−0.0008 (12)	0.0108 (12)	−0.0087 (13)
C53	0.0285 (14)	0.0356 (16)	0.0370 (17)	0.0077 (12)	−0.0008 (13)	−0.0053 (14)
C54	0.0342 (15)	0.0274 (15)	0.0401 (17)	0.0035 (12)	0.0008 (13)	−0.0063 (13)
C55	0.0295 (14)	0.0348 (16)	0.0314 (16)	0.0035 (12)	0.0037 (12)	−0.0050 (13)
C56	0.0381 (16)	0.0333 (16)	0.0326 (17)	0.0022 (13)	0.0074 (13)	0.0049 (13)
C57	0.0375 (16)	0.0280 (15)	0.0400 (17)	−0.0051 (12)	0.0142 (14)	−0.0005 (14)
O58	0.0476 (12)	0.0386 (12)	0.0419 (13)	0.0017 (9)	−0.0145 (10)	−0.0025 (10)
C59	0.0499 (19)	0.055 (2)	0.0427 (19)	0.0039 (16)	−0.0093 (16)	0.0068 (17)
Co1	0.01419 (16)	0.02480 (19)	0.02621 (19)	0.00120 (13)	0.00060 (14)	0.00083 (16)

### Geometric parameters (Å, º)

**Table:**

O1—C2	1.315 (3)	C30—H30A	0.99
O1—Co1	1.9054 (16)	C30—H30B	0.99
C2—C3	1.403 (3)	C31—C32	1.512 (4)
C2—C7	1.419 (3)	C31—H31A	0.99
C3—C4	1.365 (4)	C31—H31B	0.99
C3—H3	0.95	C32—C33	1.373 (4)
C4—C5	1.396 (4)	C32—C37	1.383 (4)
C4—H4	0.95	C33—C34	1.386 (4)
C5—C6	1.369 (4)	C33—H33	0.95
C5—H5	0.95	C34—C35	1.363 (4)
C6—C7	1.402 (4)	C34—H34	0.95
C6—H6	0.95	C35—O38	1.376 (3)
C7—C8	1.440 (4)	C35—C36	1.379 (4)
C8—N9	1.282 (3)	C36—C37	1.375 (4)
C8—H8	0.95	C36—H36	0.95
N9—C10	1.477 (3)	C37—H37	0.95
N9—Co1	1.952 (2)	O38—C39	1.428 (4)
C10—C11	1.538 (4)	C39—H39A	0.98
C10—H10A	0.99	C39—H39B	0.98
C10—H10B	0.99	C39—H39C	0.98
C11—C12	1.503 (4)	O41—C42	1.311 (3)
C11—H11A	0.99	O41—Co1	1.8955 (18)
C11—H11B	0.99	C42—C47	1.409 (4)
C12—C17	1.384 (4)	C42—C43	1.418 (4)
C12—C13	1.389 (4)	C43—C44	1.386 (4)
C13—C14	1.389 (5)	C43—H43	0.95
C13—H13	0.95	C44—C45	1.381 (5)
C14—C15	1.378 (5)	C44—H44	0.95
C14—H14	0.95	C45—C46	1.375 (5)
C15—O18A	1.378 (4)	C45—H45	0.95
C15—C16	1.384 (5)	C46—C47	1.408 (4)
C16—C17	1.389 (4)	C46—H46	0.95
C16—H16	0.95	C47—C48	1.436 (4)
C17—H17	0.95	C48—N49	1.284 (3)
O18A—C19A	1.469 (6)	C48—H48	0.95
C19A—H19A	0.98	N49—C50	1.479 (3)
C19A—H19B	0.98	N49—Co1	1.944 (2)
C19A—H19C	0.98	C50—C51	1.525 (4)
C19B—H19D	0.98	C50—H50A	0.99
C19B—H19E	0.98	C50—H50B	0.99
C19B—H19F	0.98	C51—C52	1.509 (4)
O21—C22	1.306 (3)	C51—H51A	0.99
O21—Co1	1.8791 (17)	C51—H51B	0.99
C22—C23	1.404 (4)	C52—C57	1.381 (4)
C22—C27	1.414 (3)	C52—C53	1.401 (4)
C23—C24	1.368 (4)	C53—C54	1.374 (4)
C23—H23	0.95	C53—H53	0.95
C24—C25	1.405 (4)	C54—C55	1.389 (4)
C24—H24	0.95	C54—H54	0.95
C25—C26	1.364 (4)	C55—O58	1.370 (3)
C25—H25	0.95	C55—C56	1.386 (4)
C26—C27	1.409 (4)	C56—C57	1.388 (4)
C26—H26	0.95	C56—H56	0.95
C27—C28	1.436 (4)	C57—H57	0.95
C28—N29	1.287 (3)	O58—C59	1.419 (3)
C28—H28	0.95	C59—H59A	0.98
N29—C30	1.482 (3)	C59—H59B	0.98
N29—Co1	1.9512 (19)	C59—H59C	0.98
C30—C31	1.518 (4)		
			
C2—O1—Co1	119.05 (14)	C32—C33—C34	122.1 (3)
O1—C2—C3	119.9 (2)	C32—C33—H33	118.9
O1—C2—C7	123.1 (2)	C34—C33—H33	118.9
C3—C2—C7	117.0 (2)	C35—C34—C33	119.9 (3)
C4—C3—C2	121.7 (2)	C35—C34—H34	120.1
C4—C3—H3	119.1	C33—C34—H34	120.1
C2—C3—H3	119.1	C34—C35—O38	124.2 (2)
C3—C4—C5	121.2 (3)	C34—C35—C36	119.4 (3)
C3—C4—H4	119.4	O38—C35—C36	116.4 (3)
C5—C4—H4	119.4	C37—C36—C35	119.8 (3)
C6—C5—C4	118.6 (3)	C37—C36—H36	120.1
C6—C5—H5	120.7	C35—C36—H36	120.1
C4—C5—H5	120.7	C36—C37—C32	122.1 (3)
C5—C6—C7	121.3 (3)	C36—C37—H37	119
C5—C6—H6	119.4	C32—C37—H37	119
C7—C6—H6	119.4	C35—O38—C39	116.7 (2)
C6—C7—C2	120.1 (2)	O38—C39—H39A	109.5
C6—C7—C8	119.3 (2)	O38—C39—H39B	109.5
C2—C7—C8	120.4 (2)	H39A—C39—H39B	109.5
N9—C8—C7	125.8 (2)	O38—C39—H39C	109.5
N9—C8—H8	117.1	H39A—C39—H39C	109.5
C7—C8—H8	117.1	H39B—C39—H39C	109.5
C8—N9—C10	116.5 (2)	C42—O41—Co1	120.99 (17)
C8—N9—Co1	121.44 (18)	O41—C42—C47	123.5 (2)
C10—N9—Co1	122.01 (16)	O41—C42—C43	118.8 (3)
N9—C10—C11	110.96 (19)	C47—C42—C43	117.7 (2)
N9—C10—H10A	109.4	C44—C43—C42	120.1 (3)
C11—C10—H10A	109.4	C44—C43—H43	120
N9—C10—H10B	109.4	C42—C43—H43	120
C11—C10—H10B	109.4	C45—C44—C43	121.9 (3)
H10A—C10—H10B	108	C45—C44—H44	119
C12—C11—C10	112.6 (2)	C43—C44—H44	119
C12—C11—H11A	109.1	C46—C45—C44	119.1 (3)
C10—C11—H11A	109.1	C46—C45—H45	120.5
C12—C11—H11B	109.1	C44—C45—H45	120.5
C10—C11—H11B	109.1	C45—C46—C47	120.8 (3)
H11A—C11—H11B	107.8	C45—C46—H46	119.6
C17—C12—C13	117.2 (3)	C47—C46—H46	119.6
C17—C12—C11	120.2 (3)	C46—C47—C42	120.5 (3)
C13—C12—C11	122.5 (3)	C46—C47—C48	118.4 (3)
C14—C13—C12	121.1 (3)	C42—C47—C48	121.0 (2)
C14—C13—H13	119.5	N49—C48—C47	126.1 (2)
C12—C13—H13	119.5	N49—C48—H48	116.9
C15—C14—C13	120.3 (3)	C47—C48—H48	116.9
C15—C14—H14	119.8	C48—N49—C50	117.2 (2)
C13—C14—H14	119.8	C48—N49—Co1	121.58 (19)
C14—C15—O18A	117.7 (3)	C50—N49—Co1	121.15 (16)
C14—C15—C16	119.9 (3)	N49—C50—C51	113.20 (19)
O18A—C15—C16	122.3 (4)	N49—C50—H50A	108.9
C15—C16—C17	118.7 (3)	C51—C50—H50A	108.9
C15—C16—H16	120.6	N49—C50—H50B	108.9
C17—C16—H16	120.6	C51—C50—H50B	108.9
C12—C17—C16	122.7 (3)	H50A—C50—H50B	107.8
C12—C17—H17	118.7	C52—C51—C50	110.4 (2)
C16—C17—H17	118.7	C52—C51—H51A	109.6
C15—O18A—C19A	119.8 (3)	C50—C51—H51A	109.6
H19D—C19B—H19E	109.5	C52—C51—H51B	109.6
H19D—C19B—H19F	109.5	C50—C51—H51B	109.6
H19E—C19B—H19F	109.5	H51A—C51—H51B	108.1
C22—O21—Co1	127.15 (16)	C57—C52—C53	116.9 (3)
O21—C22—C23	118.0 (2)	C57—C52—C51	122.2 (3)
O21—C22—C27	123.7 (2)	C53—C52—C51	120.8 (3)
C23—C22—C27	118.2 (2)	C54—C53—C52	121.9 (3)
C24—C23—C22	121.2 (3)	C54—C53—H53	119
C24—C23—H23	119.4	C52—C53—H53	119
C22—C23—H23	119.4	C53—C54—C55	120.0 (3)
C23—C24—C25	120.7 (3)	C53—C54—H54	120
C23—C24—H24	119.7	C55—C54—H54	120
C25—C24—H24	119.7	O58—C55—C56	125.1 (3)
C26—C25—C24	119.2 (3)	O58—C55—C54	115.5 (2)
C26—C25—H25	120.4	C56—C55—C54	119.4 (3)
C24—C25—H25	120.4	C55—C56—C57	119.7 (3)
C25—C26—C27	121.4 (3)	C55—C56—H56	120.2
C25—C26—H26	119.3	C57—C56—H56	120.2
C27—C26—H26	119.3	C52—C57—C56	122.1 (3)
C26—C27—C22	119.2 (3)	C52—C57—H57	118.9
C26—C27—C28	118.4 (2)	C56—C57—H57	118.9
C22—C27—C28	122.2 (2)	C55—O58—C59	117.4 (2)
N29—C28—C27	127.0 (2)	O58—C59—H59A	109.5
N29—C28—H28	116.5	O58—C59—H59B	109.5
C27—C28—H28	116.5	H59A—C59—H59B	109.5
C28—N29—C30	115.5 (2)	O58—C59—H59C	109.5
C28—N29—Co1	123.24 (18)	H59A—C59—H59C	109.5
C30—N29—Co1	121.19 (16)	H59B—C59—H59C	109.5
N29—C30—C31	114.6 (2)	O21—Co1—O41	174.44 (7)
N29—C30—H30A	108.6	O21—Co1—O1	84.66 (7)
C31—C30—H30A	108.6	O41—Co1—O1	89.92 (7)
N29—C30—H30B	108.6	O21—Co1—N49	89.84 (8)
C31—C30—H30B	108.6	O41—Co1—N49	91.18 (9)
H30A—C30—H30B	107.6	O1—Co1—N49	87.69 (8)
C32—C31—C30	108.3 (2)	O21—Co1—N29	93.49 (8)
C32—C31—H31A	110	O41—Co1—N29	91.96 (8)
C30—C31—H31A	110	O1—Co1—N29	177.69 (9)
C32—C31—H31B	110	N49—Co1—N29	90.93 (8)
C30—C31—H31B	110	O21—Co1—N9	90.58 (8)
H31A—C31—H31B	108.4	O41—Co1—N9	88.22 (8)
C33—C32—C37	116.7 (3)	O1—Co1—N9	90.43 (8)
C33—C32—C31	121.1 (3)	N49—Co1—N9	178.02 (8)
C37—C32—C31	122.1 (3)	N29—Co1—N9	90.98 (8)
			
Co1—O1—C2—C3	144.48 (19)	Co1—O41—C42—C43	−150.49 (18)
Co1—O1—C2—C7	−37.7 (3)	O41—C42—C43—C44	−178.8 (2)
O1—C2—C3—C4	178.6 (2)	C47—C42—C43—C44	−1.7 (4)
C7—C2—C3—C4	0.7 (4)	C42—C43—C44—C45	1.7 (4)
C2—C3—C4—C5	−0.4 (4)	C43—C44—C45—C46	−0.3 (4)
C3—C4—C5—C6	−0.7 (5)	C44—C45—C46—C47	−1.0 (4)
C4—C5—C6—C7	1.4 (5)	C45—C46—C47—C42	1.0 (4)
C5—C6—C7—C2	−1.2 (4)	C45—C46—C47—C48	176.5 (2)
C5—C6—C7—C8	−177.9 (3)	O41—C42—C47—C46	177.3 (2)
O1—C2—C7—C6	−177.8 (2)	C43—C42—C47—C46	0.4 (4)
C3—C2—C7—C6	0.1 (4)	O41—C42—C47—C48	1.9 (4)
O1—C2—C7—C8	−1.1 (4)	C43—C42—C47—C48	−175.0 (2)
C3—C2—C7—C8	176.8 (2)	C46—C47—C48—N49	169.4 (2)
C6—C7—C8—N9	−164.7 (3)	C42—C47—C48—N49	−15.1 (4)
C2—C7—C8—N9	18.5 (4)	C47—C48—N49—C50	173.8 (2)
C7—C8—N9—C10	−170.3 (2)	C47—C48—N49—Co1	−8.2 (4)
C7—C8—N9—Co1	6.1 (4)	C48—N49—C50—C51	78.8 (3)
C8—N9—C10—C11	78.4 (3)	Co1—N49—C50—C51	−99.2 (2)
Co1—N9—C10—C11	−98.0 (2)	N49—C50—C51—C52	−179.7 (2)
N9—C10—C11—C12	155.6 (2)	C50—C51—C52—C57	−100.5 (3)
C10—C11—C12—C17	−89.9 (3)	C50—C51—C52—C53	75.1 (3)
C10—C11—C12—C13	87.2 (3)	C57—C52—C53—C54	−1.2 (4)
C17—C12—C13—C14	1.5 (4)	C51—C52—C53—C54	−177.1 (2)
C11—C12—C13—C14	−175.7 (3)	C52—C53—C54—C55	−0.3 (4)
C12—C13—C14—C15	−0.6 (5)	C53—C54—C55—O58	−178.4 (2)
C13—C14—C15—O18A	177.7 (3)	C53—C54—C55—C56	1.4 (4)
C13—C14—C15—C16	−0.2 (5)	O58—C55—C56—C57	178.9 (2)
C14—C15—C16—C17	0.2 (5)	C54—C55—C56—C57	−0.9 (4)
O18A—C15—C16—C17	−177.6 (3)	C53—C52—C57—C56	1.7 (4)
C13—C12—C17—C16	−1.5 (4)	C51—C52—C57—C56	177.5 (2)
C11—C12—C17—C16	175.7 (3)	C55—C56—C57—C52	−0.7 (4)
C15—C16—C17—C12	0.7 (5)	C56—C55—O58—C59	−0.7 (4)
C14—C15—O18A—C19A	−165.3 (4)	C54—C55—O58—C59	179.1 (3)
C16—C15—O18A—C19A	12.6 (5)	C22—O21—Co1—O1	161.3 (2)
Co1—O21—C22—C23	−166.89 (19)	C22—O21—Co1—N49	−111.0 (2)
Co1—O21—C22—C27	15.6 (3)	C22—O21—Co1—N29	−20.1 (2)
O21—C22—C23—C24	−175.2 (3)	C22—O21—Co1—N9	70.9 (2)
C27—C22—C23—C24	2.5 (4)	C42—O41—Co1—O1	45.27 (19)
C22—C23—C24—C25	−0.7 (5)	C42—O41—Co1—N49	−42.42 (19)
C23—C24—C25—C26	−1.2 (5)	C42—O41—Co1—N29	−133.39 (19)
C24—C25—C26—C27	1.3 (5)	C42—O41—Co1—N9	135.70 (19)
C25—C26—C27—C22	0.5 (4)	C2—O1—Co1—O21	−43.33 (18)
C25—C26—C27—C28	175.1 (3)	C2—O1—Co1—O41	135.43 (18)
O21—C22—C27—C26	175.1 (2)	C2—O1—Co1—N49	−133.39 (19)
C23—C22—C27—C26	−2.3 (4)	C2—O1—Co1—N9	47.21 (19)
O21—C22—C27—C28	0.7 (4)	C48—N49—Co1—O21	−144.0 (2)
C23—C22—C27—C28	−176.8 (2)	C50—N49—Co1—O21	33.84 (18)
C26—C27—C28—N29	179.3 (2)	C48—N49—Co1—O41	30.5 (2)
C22—C27—C28—N29	−6.2 (4)	C50—N49—Co1—O41	−151.62 (18)
C27—C28—N29—C30	179.3 (2)	C48—N49—Co1—O1	−59.4 (2)
C27—C28—N29—Co1	−4.6 (4)	C50—N49—Co1—O1	118.51 (18)
C28—N29—C30—C31	−104.9 (3)	C48—N49—Co1—N29	122.5 (2)
Co1—N29—C30—C31	78.9 (3)	C50—N49—Co1—N29	−59.64 (18)
N29—C30—C31—C32	175.5 (2)	C28—N29—Co1—O21	14.3 (2)
C30—C31—C32—C33	−90.8 (3)	C30—N29—Co1—O21	−169.75 (18)
C30—C31—C32—C37	84.2 (3)	C28—N29—Co1—O41	−164.6 (2)
C37—C32—C33—C34	1.4 (5)	C30—N29—Co1—O41	11.36 (18)
C31—C32—C33—C34	176.7 (3)	C28—N29—Co1—N49	104.2 (2)
C32—C33—C34—C35	−0.9 (5)	C30—N29—Co1—N49	−79.86 (19)
C33—C34—C35—O38	179.5 (3)	C28—N29—Co1—N9	−76.3 (2)
C33—C34—C35—C36	−0.4 (5)	C30—N29—Co1—N9	99.61 (19)
C34—C35—C36—C37	1.0 (5)	C8—N9—Co1—O21	52.5 (2)
O38—C35—C36—C37	−178.9 (3)	C10—N9—Co1—O21	−131.27 (18)
C35—C36—C37—C32	−0.5 (5)	C8—N9—Co1—O41	−122.1 (2)
C33—C32—C37—C36	−0.7 (5)	C10—N9—Co1—O41	54.16 (18)
C31—C32—C37—C36	−175.9 (3)	C8—N9—Co1—O1	−32.2 (2)
C34—C35—O38—C39	0.3 (4)	C10—N9—Co1—O1	144.07 (18)
C36—C35—O38—C39	−179.8 (3)	C8—N9—Co1—N29	146.0 (2)
Co1—O41—C42—C47	32.7 (3)	C10—N9—Co1—N29	−37.77 (18)

### Hydrogen-bond geometry (Å, º)

Cg1 and Cg2 are the centroids of the C52–C57 and C42–C47 rings, respectively.

**Table:**

*D*—H···*A*	*D*—H	H···*A*	*D*···*A*	*D*—H···*A*
C4—H4···*Cg*1^i^	0.95	2.56	3.475 (3)	162
C30—H30*B*···*Cg*2^ii^	0.99	2.91	3.838 (3)	157

Symmetry codes: (i) −*x*−3/2, *y*, *z*−1/2; (ii) −*x*−1/2, *y*, *z*−1/2.
